# Pediatric Autoimmune Hepatitis

**DOI:** 10.3390/pediatric16010011

**Published:** 2024-02-01

**Authors:** Silvia Nastasio, Marco Sciveres, Paola Francalanci, Giuseppe Maggiore

**Affiliations:** 1 Division of Gastroenterology, Hepatology & Nutrition, Boston Children’s Hospital and Harvard Medical School, Boston, MA 02115, USA; silvia.nastasio@childrens.harvard.edu; 2 Pediatric Department and Transplantation, ISMETT, 90133 Palermo, Italy; msciveres@ismett.edu; 3 Division of Pathology, Bambino Gesù Children’s Hospital, IRCCS, 00165 Rome, Italy; paola.francalanci@opbg.net; 4 Division of Hepatogastroenterology, and Liver Transplant, ERN RARE LIVER, Bambino Gesù Children’s Hospital, IRCCS, 00165 Rome, Italy

Pediatric autoimmune hepatitis (PAIH) is a rare necro-inflammatory disease of the liver of unknown etiology thought to derive from the dysregulation of the immune response upon exposure to environmental triggers in genetically predisposed individuals. PAIH is severe and progressive and, if untreated, leads to cirrhosis and liver failure [1,2,3,4]. Two types of PAIH have been characterized based on the presence of specific circulating antibodies: PAIH type 1, in which smooth muscle antibodies (SMAs) and/or antinuclear antibodies (ANAs) are present, and PAIH type 2, which is identified using liver kidney microsomal type 1 antibodies (anti-LKM-1s) and/or anti-liver cytosol type 1 antibodies (anti-LC1s) [1]. PAIH can present with a variety of clinical scenarios, including acute hepatitis and liver failure, but it can also present with non-specific symptoms such as fatigue and abdominal pain or with an incidental finding of elevated liver enzymes or advanced liver disease (Table 1).

Once other causes of liver disease have been excluded (e.g., viral hepatitis, drug-induced liver injury, hereditary, metabolic), PAIH should be suspected in the presence of three main laboratory and histologic features: (1) hypergammaglobulinemia, (2) specific circulating autoantibodies, as described above, and (3) interface hepatitis with dense portal lymphoplasmacytic infiltrate on liver biopsy (Figure 1). 

A simplified diagnostic scoring system for AIH was developed in 2008 on the basis of the scoring system created by the International Autoimmune Hepatitis Group (IAIHG) in 1993 [5,6]. The simplified criteria show high specificity (95%) but only moderate sensitivity (77%) for the diagnosis of autoimmune hepatitis in children, making the scoring system a helpful tool although not sufficient to rule out PAIH [7]. Due to the severe and progressive nature of PAIH, immunosuppressive treatment should be started promptly at diagnosis with the goal to halt inflammation and ultimately prevent the development of cirrhosis and end-stage liver disease. First-line treatment consists of a combination of prednisolone or prednisone (1–2 mg/kg daily) in association with azathioprine (an initial dose of 1 mg/kg/day which can be further increased up to 2.5 mg/kg/day). Calcineurin inhibitors (cyclosporine and tacrolimus) and mycophenolate are the most well-known alternative therapies used in difficult-to-treat cases, such as in those with failure to induce complete remission with first-line treatment and in those with multiple relapses during tapering or discontinuation attempts [1,2,3,4]. Overall, none of these treatments can restore immune tolerance and side effects are not negligeable; however, at least multiple studies have shown high rates of remission with first-line therapy of up to 80–90%. After starting immunosuppressive treatment, patients are followed with serial blood draws which include monitoring transaminases, gamma glutamyl transferase, bilirubin, albumin, prothrombin ratio/INR, and the immunoglobulin G level, as well as the specific circulating antibodies. The aim of the treatment is to achieve complete biochemical remission, which is defined as a strict normalization of serum activities of ALT/AST and immunoglobulin G values and negative or very low-titer autoantibodies in addition to the normalization of hepatic function [1,2]. Despite the high first remission rate mentioned above, PAIH is burdened by an equally high risk of relapse reported to occur in 60–80% of children after treatment withdrawal [8,9]. Additionally, up to 8–16% of pediatric patients with PAIH have been reported to require a liver transplant, with the main indications being acute liver failure unresponsive to salvage therapy, acute chronic liver disease, complications of end-stage liver disease, and hepatocellular carcinoma [10,11].

We recently published a long-term study on a large group of 117 children with type 1 or type 2 PAIH with a 20-year median follow-up in survivors, which provided additional insight in the progression of this disease [12]. The patients in our study were mainly treated with a combination of prednisone and azathioprine, and treatment withdrawal was attempted when transaminases had been persistently normal. In a subset of patients managed before 1981, withdrawal was performed after a liver biopsy had been carried out; however, once it was noted that histologic remission did not reliably predict the risk of relapse, a biopsy was no longer considered necessary for treatment withdrawal. We found that overall, the normalization of aminotransferase was observed in more than 90% of children but particular attention needs to be paid to the prothrombin ratio as the lack of its normalization or its decline after initial normalization was found to be independently associated with a lower probability of overall and native liver survivals. The probabilities of overall and native liver survival at 30 years were 81% and 61%, respectively. It is well known that AIH is a rapidly progressive disease, and it was not surprising to see that the presence of cirrhosis, the lack of normalization of the prothrombin ratio while on treatment, gastroesophageal varices, and gastrointestinal bleeding from varices are related to a longer interval between first symptoms and diagnosis. This was especially significant since cirrhosis was present in close to half of the children seen within 3 months of the first sign of liver disease. Relapse-free treatment withdrawal ≥4 years was seen in 19% of the overall population (including patients who self-discontinued treatment) and in 42% of patients in whom withdrawal was undertaken under medical supervision. In the majority of patients, the criteria for withdrawal included persistently normal alanine aminotransferase activity without an assessment of liver histology. This finding aligns with the recent AASLD guidelines, which state that liver tissue examination is not mandatory prior to treatment withdrawal in adults and suggest that similar criteria could be applied to the pediatric population [3]. Additionally, our data did not show any difference in the outcome of treatment withdrawal under medical supervision in patients at the time of puberty compared to at other ages. This result is particularly interesting given the recommendation to avoid withdrawal attempts at the time of puberty based on the notion that hormonal changes may increase the risk of immune perturbations. Moreover, although PAIH type 2 has long been thought to be more severe than type 1, our results did not show any differences between the two types of PAIH in regard to sustained alanine aminotransferase normalization on azathioprine monotherapy, successful treatment withdrawal, and overall long-term native liver survival [12,13].

In conclusion, PAIH is a severe and rapidly progressive liver disease for which providers need to maintain a high index of clinical suspicion, expedite work when suspected, and promptly initiate treatment upon diagnosis to halt disease progression. Unfortunately, current treatments do not restore immune tolerance, but recent results offer hope of a 20% possibility of relatively long-term treatment-free survival throughout childhood and early adulthood with present-day treatments.

## Figures and Tables

**Figure 1:**
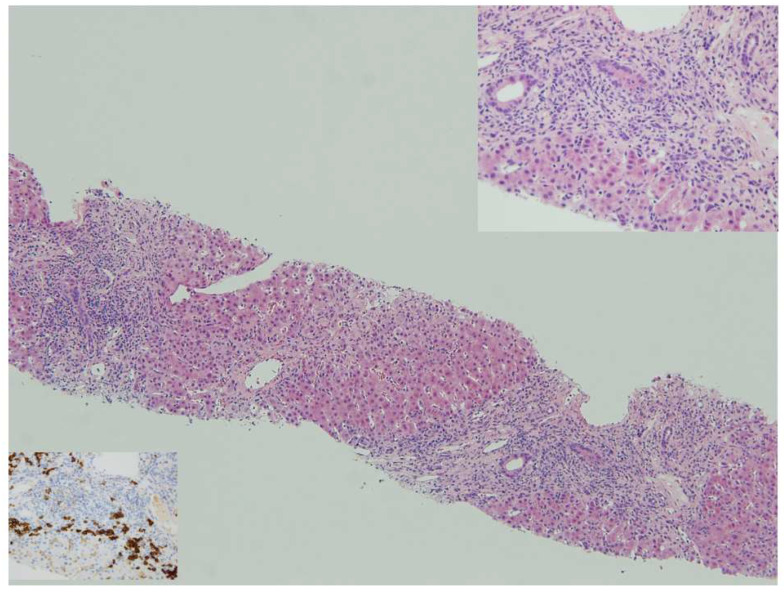
Autoimmune hepatitis: panacinar hepatitis with bridging necrosis. Lymphoplasmacytic portal and periportal infiltrates with active interface hepatitis, HE 10x. Top insert: Plasma cells in clusters are typically abundant at the interface, HE 40x. Bottom insert: anti-CD38 shows numerous plasma cells at the progression front of the inflammatory process, anti-CD38 40x.

**Table 1:** Most common presentations of JAIH.

Type of Presentation	Clinical Characteristics	Frequency	Additional Notes
Acute onset	Anorexia, nausea, vomiting, and abdominal pain followed by jaundice, similar to acute viral hepatitis.	Most common presentation	Some patients go on to develop acute liver failure with encephalopathy
Insidious onset	Progressive fatigue, anorexia, and intermittent jaundice lasting for several months/years before diagnosis	~30% of patients	All patients have clinical evidence of chronic liver disease and/or cirrhosis at diagnosis
Asymptomatic	No symptoms	~10–15% of patients	Incidental finding of clinical signs of chronic liver disease or elevated transaminases
Symptomatic portal hypertension	Variceal bleed; ascites	Rare	
JIAH presenting with symptoms related to an extrahepatic autoimmune disease	Symptoms related to autoimmune thrombocytopenia, autoimmune hemolytic anemia, type 1 diabetes, autoimmune thyroiditis, vitiligo, cutaneous vasculitis, uveitis, glomerulonephritis, juvenile chronic arthritis, systemic lupus erythematosus, Sjögren’s syndrome, celiac disease, and inflammatory bowel disease	Rare	

## Data Availability

Data sharing is not applicable to this article as no datasets were generated or analyzed during the current study.
